# Increased expression of OLFM4 and lysozyme during necrotizing enterocolitis in neonates: an observational research study

**DOI:** 10.1186/s12887-022-03260-y

**Published:** 2022-04-11

**Authors:** Sonja Diez, Marcus Renner, Veronika Bahlinger, Arndt Hartmann, Manuel Besendörfer, Hanna Müller

**Affiliations:** 1 Friedrich-Alexander-Universität Erlangen-Nürnberg (FAU), Pediatric Surgery, Department for General Surgery, University Hospital Erlangen, Loschgestraße 15, 91054 Erlangen, Germany; 2 Institute of Pathology, Heidelberg University Hospital, Im Neuenheimer Feld 224, 69120 Heidelberg, Germany; 3 Institute of Pathology, University Hospital Erlangen, Friedrich-Alexander-Universität Erlangen-Nürnberg (FAU), Krankenhausstraße 8-10, 91054 Erlangen, Germany; 4 Neonatology and Pediatric Intensive Care, Department of Pediatrics, University of Marburg, Baldingerstraße, 35033 Marburg, Germany

**Keywords:** OLFM4, Lysozyme, Necrotizing enterocolitis, Inflammation, Neonatal gastrointestinal diseases

## Abstract

**Background:**

In neonatal patients with necrotizing enterocolitis (NEC) the inflammatory response is mediated by a plurality of different proteins. The proteins olfactomedin 4 (OLFM4) and lysozyme (LYZ) are part of the intestinal mucosal defense and especially OLFM4 has rarely been evaluated in neonatal gastrointestinal diseases. The aim of this study was to analyze whether expression levels of both proteins of innate immunity, OLFM4 and lysozyme, were increased during NEC in neonates.

**Methods:**

Intestinal tissues of patients with NEC were examined with immunohistochemical staining of formalin-fixed and paraffin-embedded sections of resected tissue using antibodies against OLFM4 and lysozyme. Staining-positive tissues were semi-quantitatively scored from 0 (no staining), 1 (weak staining), 2 (moderate staining) to 3 (highly intense staining) by two individual investigators. Intestinal tissue of infants with volvulus was used as a control as other intestinal tissue without major inflammation was not available.

**Results:**

Both applied antibodies against OLFM4 showed different staining patterns with higher staining intensity of the antibody OLFM4 (D1E4M). OLFM4 (median score of the antibody OLFM4 (D1E4M): 3.0) and lysozyme (median score: 3.0) are highly expressed in intestinal and immune cells during NEC. Expression of OLFM4 and lysozyme in the control samples with volvulus was observable but significantly lower (median score of the antibody OLFM4 (D1E4M): 1.25; median score of the antibody against LYZ: 2.0; *p* = 0.033 and *p* = 0.037, respectively).

**Conclusions:**

Both proteins, OLFM4 and lysozyme, may play a role in the pathogenesis of NEC in neonatal patients, but the exact mechanisms of OLFM4 and lysozyme function and their role in immunological responses have not yet been resolved in detail. These observations add new insights as basis for further large-scale population research.

## Background

**Fig. 1:**
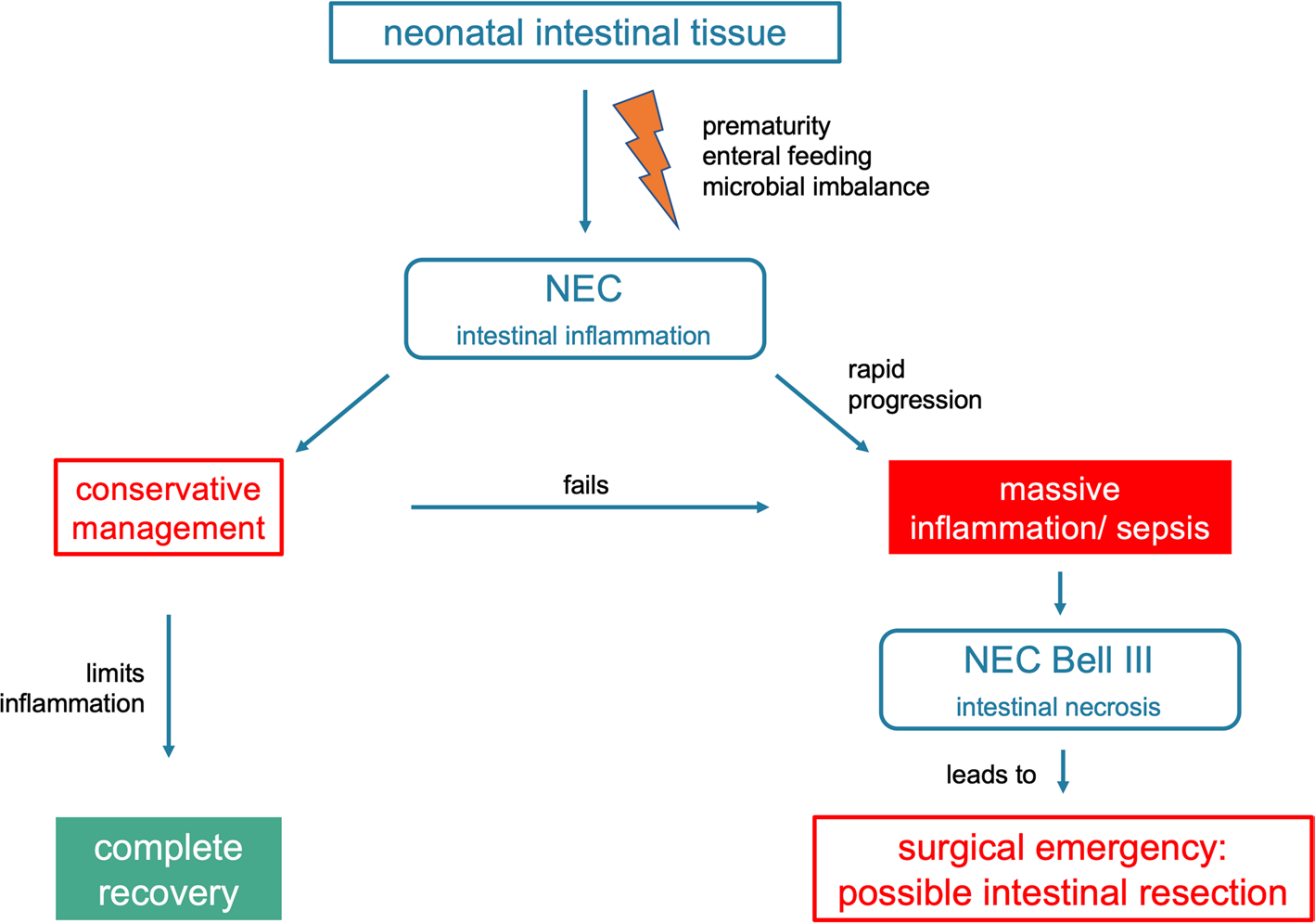
The pathogenesis of NEC. NEC triggers an intestinal inflammation. Only a small portion of patients shows a severe NEC (Bell III) with surgical indication. In these cases, intestinal necrosis demands an emergency surgery as in all patients with volvulus

Necrotizing enterocolitis (NEC) is a severe and life-threatening complication in the treatment of preterm infants [1]. It can also be diagnosed in term infants, especially with concomitant diseases such as congenital cardiac malformations [2]. It is characterized by an intestinal inflammation, which is conservatively treatable in the majority of patients. Severe cases are observable with a systemic inflammatory response with the need of intensive care medicine including mechanical ventilation, catecholamine therapy and broad antibiotic treatment. The massive inflammatory response is mediated by a plurality of different proteins as part of the intestinal innate immune system (see Fig. 1) [3]. For single proteins, such as e.g., DMBT1, the essential role in gastrointestinal diseases with a severe systemic inflammatory response has been examined [4]. Exact mechanisms, however, seem to be different. Further two of these proteins, olfactomedin 4 (OLFM4) and lysozyme (LYZ) are of major interest in the presented study.

OLFM4 is expressed under physiological conditions especially in the small intestine, colon, and prostate. It also moderately occurs in the stomach and bone marrow [5]. OLFM4 is found in macrophages, and mature human neutrophils express OLFM4 within the specific granules [6]. Its central function is determined in the mucosal defense of the stomach, small intestine and colon [7] and plays an important role in the innate immunity against bacterial infections [8]. The influence of OLFM4 was confirmed by experiments with OLFM4 deficient mice, showing a severe inflammation and proliferation in intestinal crypts of the small intestine and additional mucosal damage in the colon [9]. It is centrally discussed in pathogenesis of inflammatory bowel diseases (IBD), in which OLFM4 mRNA and protein expression levels are significantly increased in the intestinal epithelium [7, 10]. This upregulation may serve as a marker of the intestinal inflammation in IBD [8]. Most recently, TNF-α promoted OLFM4 secretion by human intestinal epithelium cells and cytoplasmic accumulation of OLFM4 in intestinal epithelium cells was observed to be promoted by Notch and TNF-α signaling, confirming a cell protective role in the inflamed mucosa of IBD patients [11]. It is additionally discussed as a robust stem cell marker for humans [12]. However, the exact regulatory mechanisms within these inflammatory conditions remain elusive and are controversially discussed [11]. In general, OLFM4 expression is highly increased during inflammatory diseases, such as sepsis, sepsis-induced acute respiratory distress syndrome, and respiratory syncytial virus infection [13–15]. In summary, OLFM4 binds to various proteins in inflammation signaling pathways and has an essential role in the innate immunity against bacterial infections, gastrointestinal inflammation, and cancer [8].

LYZ is a protein with antimicrobial and immune modulating characteristics. Its central role in the innate immunity has been proven [16]. It can be detected in different hematological cell types (macrophages, neutrophils, dendritic cells), and in the liver as well as in different secretions (saliva, urine, tears) and at mucosal surfaces. In parallel to OLMF4, LYZ influences a pro-inflammatory response and the resolution of inflammation at mucosal sites [16]. Within the first mentioned function, LYZ can hydrolyze cell wall peptidoglycan in bacteria, leading to bacterial destruction, which thereby activates pattern recognition receptors in host cells. In contrast, LYZ assists in the intestinal epithelial barrier protection to limit the invasion of the microbiota and contributes to the immune cell response in mice to resolve mucosal inflammation and to limit intestinal as well as systemic inflammation [17].

As both proteins play a substantial role in innate immunity and in the inflammatory response of intestinal tissue, we conducted the presented study evaluating the hypothesis of increased levels of both, OLMF4 and LYZ, during NEC in neonates.

## Methods and patients

### Clinical data

Intestinal tissues of seven patients with NEC were examined. Patients’ demographical data were collected retrospectively by evaluation of medical reports of the University Hospital Heidelberg. The study was approved by the local Ethics Committee of the University of Heidelberg and the local Ethics Committee of the University of Erlangen-Nürnberg and was in accordance with the Helsinki declaration (1964) and its later amendments. Gestational age was defined as time elapsed between the first day of the last menstrual period and the day of delivery. The resected intestinal tissues were exclusively gained in cases with a clinical indication for surgery due to NEC. Surgery was conducted in the acute state of the disease and during massive deterioration in severe NEC cases. In the cases of multiple surgery only sections of intestinal tissue with acute NEC were stained. The samples included tissue sections from the periphery of the active inflammatory lesions as well as the areas of necrosis or perforation and were immediately preserved in formalin to allow exact histologic assessment. We used intestinal tissue resected from patients with volvulus as control tissue with similar gestational age.

### Immunohistochemical analyses

The immunohistochemical staining of formalin-fixed and paraffin-embedded serial intestinal sections of tissue resected during NEC or volvulus were performed at the Institute of Pathology, University Hospital Heidelberg, Germany. The antigen retrieval was carried out with citrate buffer pH 6.1 (DAKO, Agilent, Waldbronn, Germany). Anti-OLFM4 antibody ab188812 (abcam, Berlin, Germany; rabbit polyclonal antibody against a synthetic 16 amino acid peptide of OLFM4) in a dilution of 1:100 and OLFM4 (D1E4M) XP® Rabbit mAb #14369 (Cell Signalling Technology, Frankfurt/Main, Germany; monoclonal antibody produced by immunizing animals with a synthetic peptide corresponding to residues surrounding Phe94 of human OLFM4 protein recognizing endogenous levels of total OLFM4 protein) in a dilution of 1:200 was used. We used these two antibodies with different targets to confirm OLFM4 expression with potentially different length or potential modifications in neonatal tissue, as data on intestinal OLFM4 in these patients are still not available. LYZ was detected in intestinal tissue using Anti-LYZ antibody (BGN/06/961, ab36362, abcam, Berlin, Germany) in a dilution of 1:100.

Staining-positive tissues were scored semi-quantitatively from 0 (no staining), 1 (weak staining), 2 (moderate staining) to 3 (highly intense staining) and each section was scored independently by two investigators (SD, HM). The average score was taken for statistics.

### Statistical analyses

Data were analyzed using the Mann-Whitney-U-test. *P*-values < 0.05 were regarded as statistically significant.

## Results

### Patients’ demographical data

The median birth weight of the seven infants with NEC was 800 g (range: 500–2940 g) and the median gestational age at birth was 26.0 weeks (range: 23.3–37.4 weeks). Surgery was done due to acute and high-staged NEC at a median postnatal age of 12 days (range: day 8–33 of life) and a median corrected gestational age of 29.6 weeks (range: 24.4–39.1 weeks). The 6 control infants with volvulus showed a median birth weight of 688 g (range: 360–1960 g) and a median gestational age at birth of 24.5 weeks (range: 22.7–33.9 weeks). Surgeries due to volvulus were conducted in an emergency setting at a median day 43 of life (range: day 2–70 of life) and at a median corrected gestational age of 33.6 (range: 24.2–34.9 weeks). Table 1 summarizes the patients’ demographic baseline data.

**Table 1:** Demographic data of participating patients

	Diagnosis	Birth weight [g]	Gestational age at birth [weeks]	Postnatal day at surgery [days]	Corrected gestational age at surgery [weeks]
1	NEC	500	23.3	8	24.4
2	NEC	1490	31.7	11	33.3
3	NEC	800	26.0	24	29.6
4	NEC	650	24.4	30	28.7
5	NEC	2940	37.4	12	39.1
6	NEC	1630	29.7	33	34.4
7	NEC	630	23.7	11	25.3
8	Control with volvulus	570	22.7	12	24.4
9	Control with volvulus	1960	33.9	2	34.1
10	Control with volvulus	740	25.0	69	34.9
11	Control with volvulus	950	27.9	48	34.7
12	Control with volvulus	635	23.1	70	33.1
13	Control with volvulus	360	24.0	38	29.4

*Abbreviations*: *NEC* Necrotizing enterocolitis

### OLFM4 and LYZ expression during NEC

**Fig. 2:**
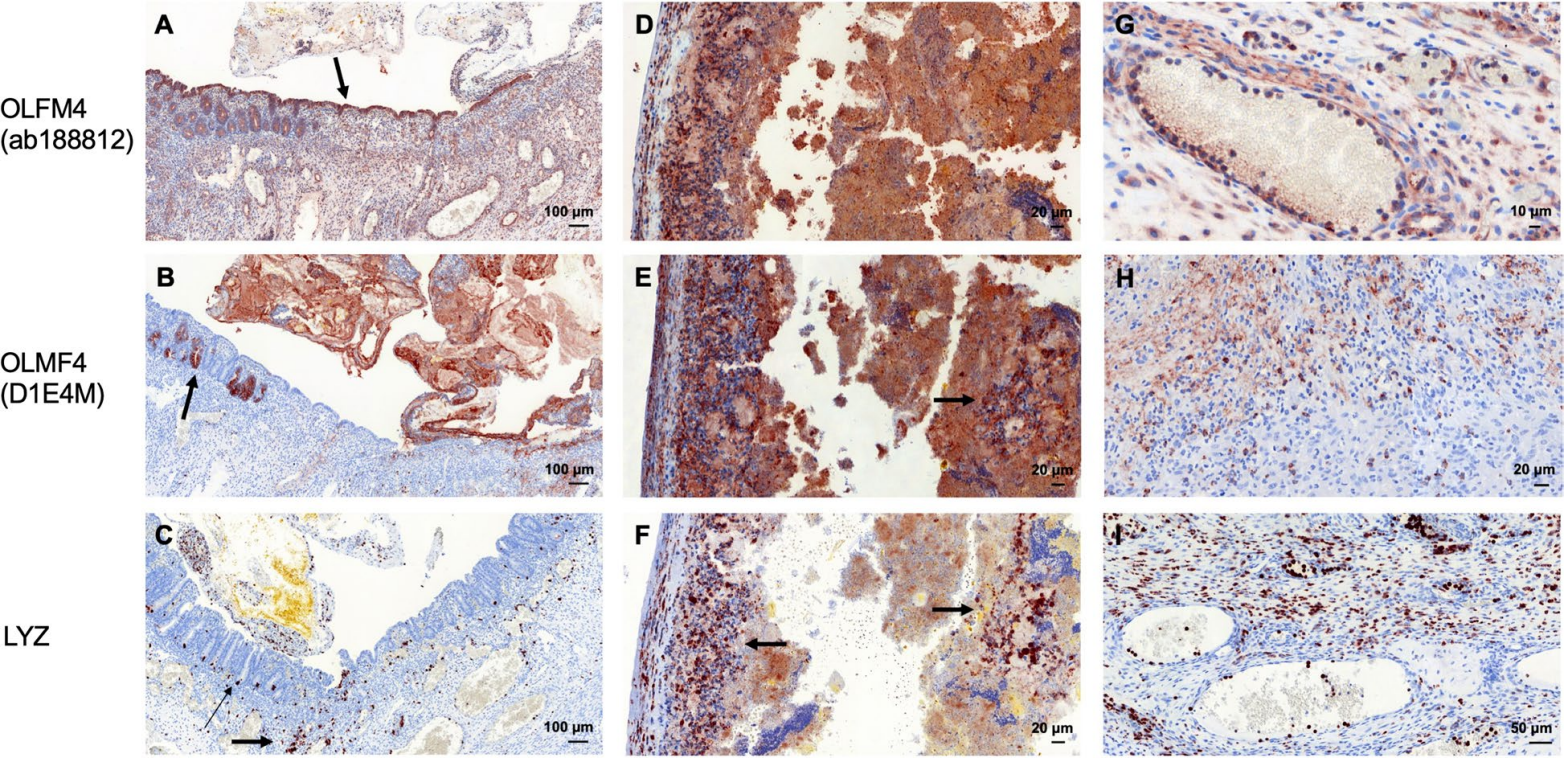
Expression of OLFM4 and LYZ in NEC tissue. **A**-**C** NEC in a preterm infant (gestational age at birth: 26 weeks, birth weight: 800 g). OLFM4 was highly expressed in the crypts (arrow in **B**) and in the intestinal epithelial cells (arrow in **A**) and in immune cells, dispersed in the whole intestinal wall (**A**). LYZ was expressed in Paneth cells of the crypts (thin arrow in **C**) and in immune cells (e.g., macrophages, thick arrow in **C**). **D**-**F** NEC in a preterm infant (gestational age at birth: 31 weeks; birth weight: 1490 g). Massive expression of OLFM4 and extensive OLFM4 and LYZ staining of macrophages (arrows in **E** & **F**). **G**-**I** Expression of OLFM4 and LYZ in immune cells during NEC. **G** OLFM4 positive immune cells attached to the endothelial cells of a large vessel in the intestinal wall. **H** Neutrophils with expression of OLFM4 located in the intestinal wall. **I** LYZ-positive immune cells in the intestinal wall migrated from vessels into intramural tissue

**Fig. 3:**
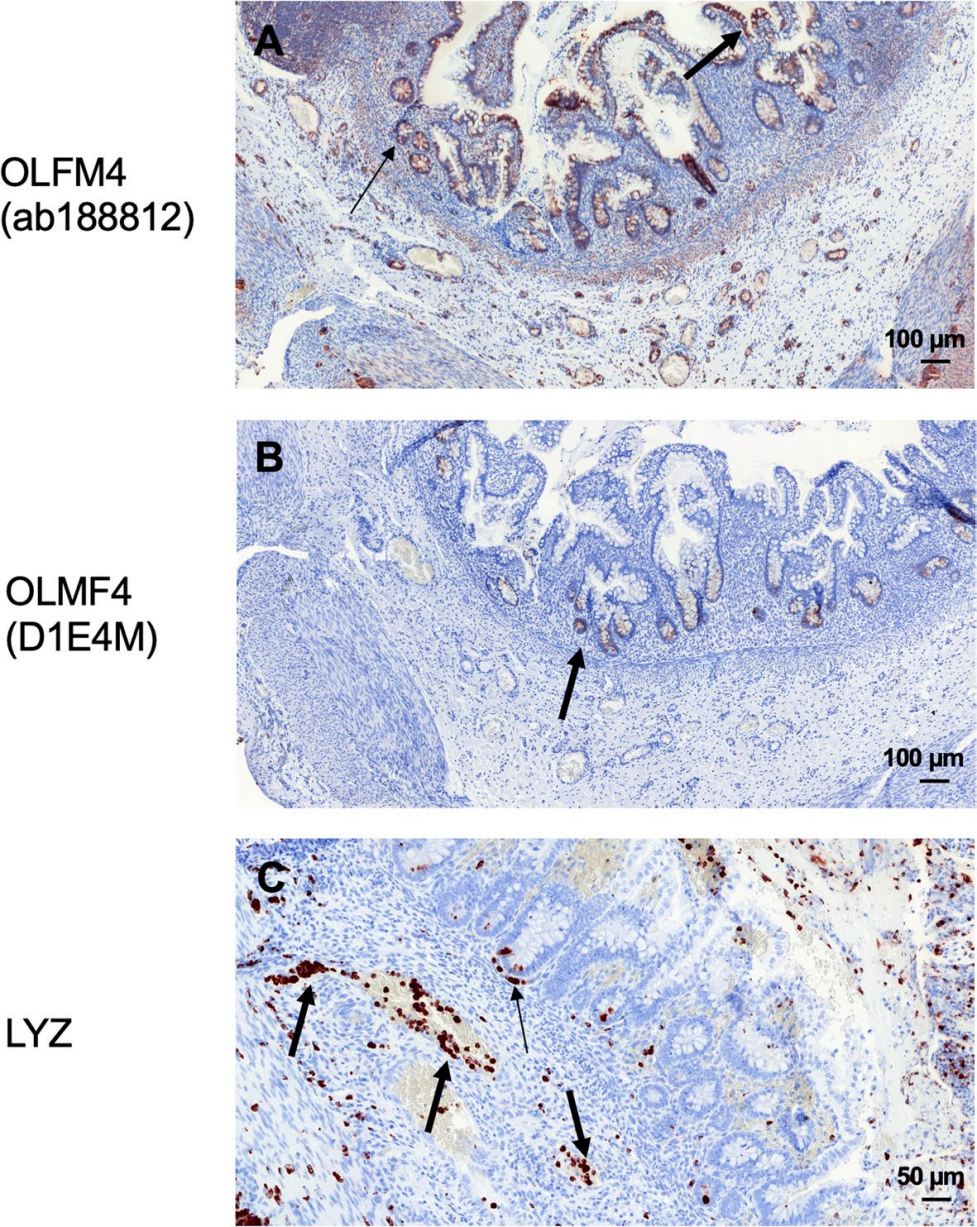
Expression of OLFM4 and LYZ in controls (intestinal tissue with volvulus). **A**, **B** OLFM4 was clearly expressed in the crypts (thin arrow in **A**, arrow in **B**) and in the intestinal epithelial cells (thick arrow in **A**). **C** Paneth cells at the base of a crypt showed expression of LYZ. Immune cells (e.g., neutrophils) also expressed LYZ (**C**), invading the crypts (thin arrow in **C**) after leaving the vessels (thick arrows in **C**)

**Fig. 4:**
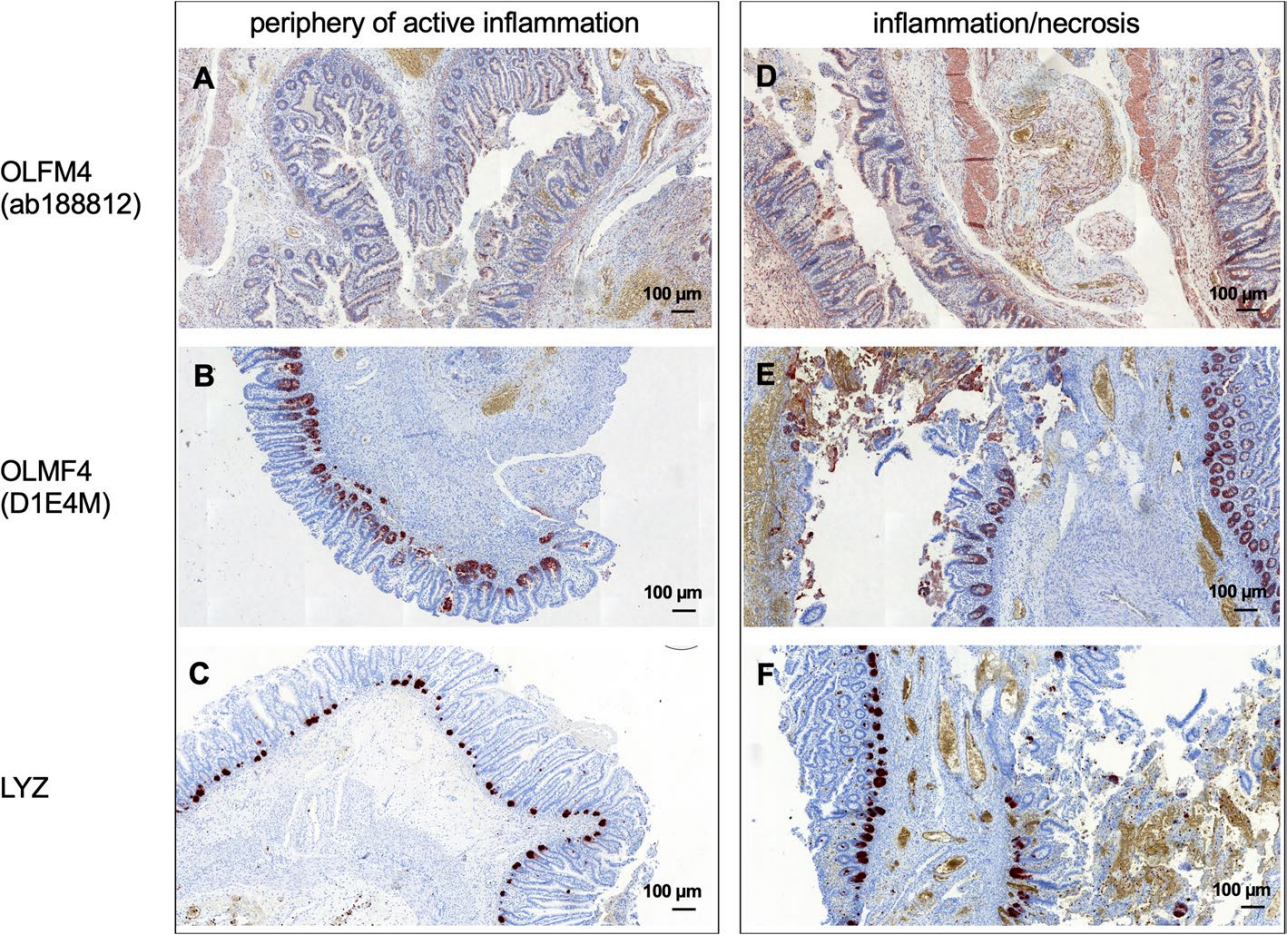
The degree of inflammation in correlation with the staining pattern and intensity. **A**-**C** demonstrate the periphery of active inflammation and **D**-**F** show regions of massive inflammation and necrosis. Expression of OLFM4 and LYZ was higher and included more intestinal epithelial and immune cells in regions with high inflammation and necrosis in comparison to regions in the periphery of active inflammation

Both applied antibodies against OLFM4 showed partially different staining patterns with higher staining intensity of the antibody OLFM4 (D1E4M). High expression of OLFM4 and LYZ was observed in the infants with NEC (median score of the antibody OLFM4 (D1E4M): 3.0 and LYZ: 3.0). Expression of OLFM4 and LYZ was higher in patients with NEC in comparison to control infants suffering from volvulus (median score of the antibody OLFM4 (D1E4M): 1.25 and lysozyme: 2.0). The difference in staining between infants with NEC and control infants with volvulus was proven statistically significant (OLFM4 (D1E4M): *p* = 0.033, LYZ: *p* = 0.037; see Figs. 2 and 3; Table 2). OLFM4 expression was observed in intestinal epithelial cells as well as in immune cells (neutrophils, macrophages) of the affected intestine in cases with NEC that were present due to severe inflammation (Fig. 2). The degree of inflammation correlated with the staining intensity and a positive correlation between inflammation and staining intensity demonstrated by an increased amount of stained intestinal epithelial as well as immune cells was observed (Fig. 4). This increase in expression in areas with high inflammatory reaction was seen in both, intestinal and colonic tissue. Furthermore, OLFM4 was detected within the mucus (Fig. 2A, B). Lysozyme expression was also high in NEC manifestation. Immune cells (neutrophils, macrophages) showed intensive LYZ expression (Fig. 2C, I). Figure 2 illustrates the expression of OLFM4 (Fig. 2G, H) and LYZ (Fig. 2I) of neutrophils in the vessels of inflamed tissue and points to the source of staining-positive immune cells migrating from the vessel into intramural tissue.

**Table 2:** Description of each section concerning OLFM4 and LYZ staining

	Diagnosis	OLFM4 staining (ab188812)	OLFM4 staining (D1E4M)	Lysozyme staining
1	NEC	1 (immune cells)	3 (immune cells)	3 (immune cells)
2	NEC	3 (immune cells)	3 (immune cells)	3 (immune cells)
3	NEC	3 (intestinal epithelial cells, immune cells)	3 (intestinal epithelial cells, immune cells)	2 (immune cells, a few crypts)
4	NEC	2.5 (intestinal epithelial cells, multiple immune cells)	3 (intestinal epithelial cells, a few immune cells)	3 (many crypts, a few immune cells)
5	NEC	3 (immune cells, intestinal epithelial cells)	3 (immune cells, intestinal epithelial cells)	2.5 (immune cells and crypts)
6	NEC	2.5 (immune cells)	3 (immune cells)	3 (immune cells)
7	NEC	1.5 (immune cells)	Adequate tissue was not available	0.5 (immune cells)
8	Control with volvulus	3 (immune cells, a few intestinal epithelial cells in the crypts)	3 (immune cells, a few intestinal epithelial cells in the crypts)	2 (immune cells, a few crypts)
9	Control with volvulus	1 (immune cells)	0.5 (immune cells)	2 (immune cells)
10	Control with volvulus	0.5 (immune cells)	1 (immune cells)	0.5 (immune cells)
11	Control with volvulus	2.5 (immune cells)	3 (immune cells, intestinal epithelial cells)	2.5 (immune cells)
12	Control with volvulus	2.5 (immune cells, intestinal epithelial cells)	1 (intestinal epithelial cells)	2.0 (crypts, a few immune cells)
13	Control with volvulus	1.5 (immune cells, a few intestinal epithelial cells in the crypts)	1.5 (immune cells, a few intestinal epithelial cells in the crypts)	0.5 (crypts, immune cells)

*Abbreviations*: *NEC* Necrotizing enterocolitis, *OLFM4* Olfactomedin 4

## Discussion

We are presenting a study on the expression of OLFM4 and LYZ in neonatal patients with NEC, confirming the hypothesis of elevated protein levels in intestinal tissue of these patients. A pronounced expression of these proteins could be observed in patients with NEC in dependence of inflammation and necrosis.

Especially in NEC, immunological aspects play a central role in current research, aiming at the improvement of knowledge on inflammatory processes and the identification of potential new therapeutic targets. Recent studies confirm a lack of knowledge in this field while highlighting the interference of mechanisms of the innate and the adaptive immune system with its deficiencies in preterm and term neonates [18–20]. Both proteins, OLFM4 and LYZ, are proven to be a part of the innate immune system. However, associations of OFLM4 to NEC have rarely been evaluated so far.

The role of OLFM4 in gastrointestinal diseases, such as IBD and gastric or colorectal cancer, has been confirmed in a plurality of publications [5], supporting its influence as sepsis regulator and confirming its survival benefit. As mentioned before, a cell protective role in the inflamed mucosa of IBD patients could be seen, regulated via the NF-kB pathway [7]. This was additionally seen in OLFM4 deficient mice with severe inflammation and proliferation in intestinal crypts of the small intestine and additional mucosal damage in the colon [9]. On the contrary, a subset of neutrophils expressing OLFM4 has been identified. Alder et al. confirmed an association of these OLFM4 neutrophils with a worse outcome during sepsis [14]. Moreover, Levinsky et al. observed that OLFM4 null mice are protected from death during sepsis and showed less intestinal barrier dysfunction, implicating that OLFM4 might contribute essential pathogenic aspects to the inflammatory process [21]. Furthermore, they described the central involvement of OLFM4-positive neutrophils in the pathologic process leading to intestinal damage and mortality after intestinal ischemia/reperfusion injury. The authors hypothesized a potential mechanism for this observation, in which activated neutrophils next to the injury secrete OLFM4 into the environment leading to enhanced iNOS production by macrophages and injured tissue. This leads to an impaired intestinal barrier [21]. We observed a moderate/high expression of OLFM4 in affected intestinal tissue and in immune cells of inflammatory intestinal regions in cases of NEC and are therefore able to support these observations. Taken together, increased OLFM4 expression is necessary in inflammation signaling pathways and has an essential role in the innate immunity against bacterial invasion, gastrointestinal inflammation, and in survival support [8]. But in cases with overwhelming inflammation and sepsis, OLFM4 switches from the cell-protective role to its predominantly pro-inflammatory role. It then loses its positive influence as sepsis regulator, leading to an impaired intestinal barrier [21].

The two different antibodies against OLFM4 showed in part different staining scores and this may have different causes: One possibility includes the presence of different OLFM protein variants as the antibodies had different targets within the protein. Furthermore, potential modifications might lead to changes at antibody binding regions and might thereby influence the staining. However, this must be analyzed by further studies.

LYZ has been evaluated regarding its role in the pathogenesis of NEC. Coutinho et al. confirmed an absence of lysozyme in Paneth cells of NEC patients compared to matched controls [22]. Furthermore, expression of LYZ was observed in macrophages of the lamina propria. They hypothesized that the loss of Paneth cells enhances infections by the impairment of the mucosal barrier [22]. Further research by McElroy et al. supported this hypothesis: a decreased Paneth cell number was observed in infants with surgically treated NEC compared to age-matched controls with spontaneous intestinal perforation [23]. The loss of Paneth cells in different murine models resulted in a NEC-like pathology in the small intestine of immature mice [24, 25]. Markasz et al. contributed further to the pathophysiological mechanism describing the absence of alpha-defensins in Paneth cells as a central factor in the pathogenesis of NEC [26]. Our study contradicts the reduced LYZ expression, as we observed an obvious expression of LYZ in NEC. This expression includes additional cells aside Paneth cells in regions of proximity to affected intestinal regions with NEC. Schaart et al. described a weak expression of LYZ in Paneth cells, but explained a possible cause for different LYZ expression levels in NEC tissue: Paneth cells rapidly secreted lysozyme in response to epithelial injury to prevent translocation of bacteria and it depends on the timepoint whether low or elevated LYZ expression levels were seen [27]. The absence of LYZ-positive Paneth cells reported in studies (e.g. [22]) might therefore be explained as a result of increased LYZ secretion [28]. Other studies described that Paneth cell antimicrobials were increased in response to NEC [29, 30]. Puiman et al. reported increased Paneth cell numbers and expression of Paneth cell antimicrobials only during recovery from NEC [28]. Despite the observation that antimicrobial proteins were up-regulated in response to NEC, the protein expression levels are low and may be inadequate to protect the immature gut from a bacterial invasion [30]. Surgical intervention within our study was early during NEC progress and removed intestinal tissue which was strongly affected or not vital during surgery. In these very strongly affected regions during early NEC process, we observed the up-regulation of OLFM4 and LYZ in intestinal epithelial cells and immune cells. Intestinal sections included tissue with higher distance to very strongly affected regions showing low expression of OLFM4 in different cells. Furthermore, the intestinal tissue far away from the very strongly affected regions demonstrated a LYZ expression in a decreased number of immune cells as well as a LYZ expression restricted to Paneth cells compared to affected regions. We assume that the different surgical time-points and different grades of destroyed and inflamed intestinal tissue may explain the contradictory findings of the studies. LYZ is described in both, up- and downregulation during inflammation. Even a certain strengthening of the mucus barrier could be observed in rat pups fed with LYZ after exposition to NEC-stressors [31]. Unfortunately, this innate defense of LYZ was not sufficient to protect the intestine.

Blood monocyte count is low in NEC and intestinal inflammatory response is rich in macrophages. Furthermore, differentiation of monocytes into intestinal macrophages and up-regulation of monocyte recruitment genes were induced by NEC [32]. We observed extensive OLFM4 and LYZ staining of macrophages in intestinal tissue of infants with NEC. This emphasizes the antimicrobial function of OLFM4 and LYZ during NEC.

Conclusively, this may lead to the hypothesis of a protective role of LYZ and OLFM4 in the development of NEC. High expression levels of both proteins may support the determination of intestinal inflammation as a local disease. Consecutively, a complete bowel recovery might be possible. Low expression levels, on the contrary, open the way to a systemic inflammatory response, resulting in an irreversible bowel necrosis and sepsis of affected children. In the case of OLFM4, the expression of OLFM4 is no longer beneficial in overwhelming inflammatory processes. OLFM4 is then involved in the destruction of the intestine. Clinical research is focusing on the role of LYZ as a potential biomarker in feces and blood of intestinal inflammatory diseases [33] and its medical application, using the protein’s anti-inflammatory, anti-bacterial and immunomodulating effects in gastroenterological diseases [34, 35].

Limitations of our study can especially be discussed regarding the small samples size and regarding timing of surgical resections. As mentioned above, different surgical time points might be responsible for differences of protein levels within the study, although all infants were timely operated within a close time window. Different methods were evaluated to serve as controls of intestinal tissue. Within the conception of the study’s design, we carefully considered further options of intestinal tissue as controls. As tissue of controls in the same age was not achievable (e.g., from patients with Hirschsprung’s disease of small bowel atresia) via bio-databanks or healthy neonates, we studied intestinal tissue from deceased neonates (for reasons other than intestinal disease). However, the epithelial structure was destroyed, and staining was not possible because of the extended time between death and autopsy. We decided to use tissue from patients with volvulus as control tissue, accepting that the possible inflammatory response in this second-best option might impede the conclusion of our study. However, we were able to compare protein levels in same-age patients.

## Conclusions

In conclusion, OLFM4 and LYZ were highly expressed in intestinal and immune cells during NEC and were associated with inflammation and necrosis. This may be especially important to limit the intestinal inflammatory progress. However, the exact mechanisms of OLFM4 and LYZ function and their role in immunological responses have not yet been resolved. These observations of an observational research study add new insights as basis for further large-scale population research and potential new therapeutic targets such as daily supplementation of LYZ [31, 36].

## Data Availability

All data generated or analyzed during this study are included in this published article.

## Abbreviations

IBD: Inflammatory bowel disease
LYZ: Lysozyme
NEC: Necrotizing enterocolitis
OLFM4: Olfactomedin 4

## References

[1] Knell J, Han SM, Jaksic T, Modi BP (2019). Current status of necrotizing enterocolitis. Curr Probl Surg.

[2] Kessler U, Hau EM, Kordasz M, Haefeli S, Tsai C, Klimek P, Cholewa D, Nelle M, Pavlovic M, Berger S (2018). Congenital heart disease increases mortality in neonates with necrotizing enterocolitis. Front Pediatr.

[3] Bein A, Eventov-Friedman S, Arbell D, Schwartz B (2018). Intestinal tight junctions are severely altered in NEC preterm neonates. Pediatr Neonatol.

[4] Muller H, Renner M, Helmke BM, Mollenhauer J, Felderhoff-Muser U (2016). Elevated DMBT1 levels in neonatal gastrointestinal diseases. Histochem Cell Biol.

[5] Wang XY, Chen SH, Zhang YN, Xu CF (2018). Olfactomedin-4 in digestive diseases: a mini-review. World J Gastroenterol.

[6] Clemmensen SN, Bohr CT, Rørvig S, Glenthøj A, Mora-Jensen H, Cramer EP, Jacobsen LC, Larsen MT, Cowland JB, Tanassi JT (2012). Olfactomedin 4 defines a subset of human neutrophils. J Leukoc Biol.

[7] Gersemann M, Becker S, Nuding S, Antoni L, Ott G, Fritz P, Oue N, Yasui W, Wehkamp J, Stange EF (2012). Olfactomedin-4 is a glycoprotein secreted into mucus in active IBD. J Crohns Colitis.

[8] Liu W, Rodgers GP (2016). Olfactomedin 4 expression and functions in innate immunity, inflammation, and cancer. Cancer Metastasis Rev.

[9] Liu W, Li H, Hong SH, Piszczek GP, Chen W, Rodgers GP (2016). Olfactomedin 4 deletion induces colon adenocarcinoma in Apc(Min/+) mice. Oncogene.

[10] Shinozaki S, Nakamura T, Iimura M, Kato Y, Iizuka B, Kobayashi M, Hayashi N (2001). Upregulation of Reg 1alpha and GW112 in the epithelium of inflamed colonic mucosa. Gut.

[11] Kuno R, Ito G, Kawamoto A, Hiraguri Y, Sugihara HY, Takeoka S, Nagata S, Takahashi J, Tsuchiya M, Anzai S (2021). Notch and TNF-α signaling promote cytoplasmic accumulation of OLFM4 in intestinal epithelium cells and exhibit a cell protective role in the inflamed mucosa of IBD patients. Biochem Biophys Rep.

[12] van der Flier LG, Haegebarth A, Stange DE, van de Wetering M, Clevers H (2009). OLFM4 is a robust marker for stem cells in human intestine and marks a subset of colorectal cancer cells. Gastroenterology.

[13] Brand HK, Ahout IML, de Ridder D, van Diepen A, Li Y, Zaalberg M, Andeweg A, Roeleveld N, de Groot R, Warris A (2015). Olfactomedin 4 serves as a marker for disease severity in pediatric respiratory syncytial virus (RSV) infection. PLoS One.

[14] Alder MN, Opoka AM, Lahni P, Hildeman DA, Wong HR (2017). Olfactomedin-4 is a candidate marker for a pathogenic neutrophil subset in septic shock. Crit Care Med.

[15] Stark JE, Opoka AM, Mallela J, Devarajan P, Ma Q, Levinsky NC, Stringer KF, Wong HR, Alder MN (2020). Juvenile OLFM4-null mice are protected from sepsis. Am J Physiol Renal Physiol.

[16] Ragland SA, Criss AK (2017). From bacterial killing to immune modulation: recent insights into the functions of lysozyme. PLoS Pathog.

[17] Markart P, Faust N, Graf T, Na CL, Weaver TE, Akinbi HT (2004). Comparison of the microbicidal and muramidase activities of mouse lysozyme M and P. Biochem J.

[18] Cho SX, Berger PJ, Nold-Petry CA, Nold MF (2016). The immunological landscape in necrotising enterocolitis. Expert Rev Mol Med.

[19] Sampah MES, Hackam DJ (2020). Dysregulated mucosal immunity and associated pathogeneses in preterm neonates. Front Immunol.

[20] Hackam DJ, Sodhi CP, Good M (2019). New insights into necrotizing enterocolitis: from laboratory observation to personalized prevention and treatment. J Pediatr Surg.

[21] Levinsky NC, Mallela J, Opoka AM, Harmon K, Lewis HV, Zingarelli B, Wong HR, Alder MN (2019). The olfactomedin-4 positive neutrophil has a role in murine intestinal ischemia/reperfusion injury. FASEB J.

[22] Coutinho HB, da Mota HC, Coutinho VB, Robalinho TI, Furtado AF, Walker E, King G, Mahida YR, Sewell HF, Wakelin D (1998). Absence of lysozyme (muramidase) in the intestinal Paneth cells of newborn infants with necrotising enterocolitis. J Clin Pathol.

[23] McElroy SJ, Prince LS, Weitkamp J-H, Reese J, Slaughter JC, Polk DB (2011). Tumor necrosis factor receptor 1-dependent depletion of mucus in immature small intestine: a potential role in neonatal necrotizing enterocolitis. Am J Physiol Gastrointest Liver Physiol.

[24] Lueschow SR, McElroy SJ (2020). The Paneth cell: the curator and defender of the immature small intestine. Front Immunol.

[25] Zhang C, Sherman MP, Prince LS, Bader D, Weitkamp JH, Slaughter JC, McElroy SJ (2012). Paneth cell ablation in the presence of Klebsiella pneumoniae induces necrotizing enterocolitis (NEC)-like injury in the small intestine of immature mice. Dis Model Mech.

[26] Markasz L, Wanders A, Szekely L, Lilja HE (2018). Diminished DEFA6 expression in Paneth cells is associated with necrotizing enterocolitis. Gastroenterol Res Pract.

[27] Schaart MW, de Bruijn AC, Bouwman DM, de Krijger RR, van Goudoever JB, Tibboel D, Renes IB (2009). Epithelial functions of the residual bowel after surgery for necrotising enterocolitis in human infants. J Pediatr Gastroenterol Nutr.

[28] Puiman PJ, Burger-van Paassen N, Schaart MW, de Bruijn ACJM, de Krijger RR, Tibboel D, van Goudoever JB, Renes IB (2011). Paneth cell hyperplasia and metaplasia in necrotizing enterocolitis. Pediatr Res.

[29] Salzman NH, Polin RA, Harris MC, Ruchelli E, Hebra A, Zirin-Butler S, Jawad A, Martin Porter E, Bevins CL (1998). Enteric defensin expression in necrotizing enterocolitis. Pediatr Res.

[30] Underwood MA, Kananurak A, Coursodon CF, Adkins-Reick CK, Chu H, Bennett SH, Wehkamp J, Castillo PA, Leonard BC, Tancredi DJ (2012). Bifidobacterium bifidum in a rat model of necrotizing enterocolitis: antimicrobial peptide and protein responses. Pediatr Res.

[31] Lock JY, Carlson TL, Yu Y, Lu J, Claud EC, Carrier RL (2020). Impact of developmental age, necrotizing enterocolitis associated stress, and oral therapeutic intervention on mucus barrier properties. Sci Rep.

[32] Managlia E, Liu SXL, Yan X, Tan XD, Chou PM, Barrett TA, De Plaen IG (2019). Blocking NF-κB activation in Ly6c(+) monocytes attenuates necrotizing enterocolitis. Am J Pathol.

[33] Di Ruscio M, Vernia F, Ciccone A, Frieri G, Latella G (2017). Surrogate fecal biomarkers in inflammatory bowel disease: rivals or complementary tools of fecal calprotectin?. Inflamm Bowel Dis.

[34] Ferraboschi P, Ciceri S, Grisenti P (2021). Applications of lysozyme, an innate immune defense factor, as an alternative antibiotic. Antibiotics.

[35] Jiang L, Li Y, Wang L, Guo J, Liu W, Meng G, Zhang L, Li M, Cong L, Sun M (2021). Recent insights into the prognostic and therapeutic applications of lysozymes. Front Pharmacol.

[36] Cheng WD, Wold KJ, Benzoni NS, Thakwalakwa C, Maleta KM, Manary MJ, Trehan I (2017). Lactoferrin and lysozyme to reduce environmental enteric dysfunction and stunting in Malawian children: study protocol for a randomized controlled trial. Trials.

